# Trends in Patient Volume by Hospital Type and the Association of These Trends With Time to Cancer Treatment Initiation

**DOI:** 10.1001/jamanetworkopen.2021.15675

**Published:** 2021-07-09

**Authors:** Zachary A. K. Frosch, Nicholas Illenberger, Nandita Mitra, Daniel J. Boffa, Matthew A. Facktor, Heidi Nelson, Bryan E. Palis, Justin E. Bekelman, Lawrence N. Shulman, Samuel U. Takvorian

**Affiliations:** 1 Division of Hematology & Oncology, Perelman School of Medicine, University of Pennsylvania, Philadelphia; 2 Penn Center for Cancer Care Innovation, Abramson Cancer Center, University of Pennsylvania, Philadelphia; 3 Leonard Davis Institute of Health Economics, University of Pennsylvania, Philadelphia; 4 Department of Biostatistics, Epidemiology & Informatics, Perelman School of Medicine, University of Pennsylvania, Philadelphia; 5 Section of Thoracic Surgery, Department of Surgery, Yale School of Medicine, New Haven, Connecticut; 6 Department of Thoracic Surgery, Geisinger Heart Institute, Danville, Pennsylvania; 7 Cancer Programs, American College of Surgeons, Chicago, Illinois; 8 Department of Radiation Oncology, Perelman School of Medicine, University of Pennsylvania, Philadelphia; 9 Department of Medical Ethics and Health Policy, Perelman School of Medicine, University of Pennsylvania, Philadelphia

## Abstract

**Importance:**

Increasing demand for cancer care may be outpacing the capacity of hospitals to provide timely treatment, particularly at referral centers such as National Cancer Institute (NCI)–designated and academic centers. Whether the rate of patient volume growth has strained hospital capacity to provide timely treatment is unknown.

**Objective:**

To evaluate trends in patient volume by hospital type and the association between a hospital’s annual patient volume growth and time to treatment initiation (TTI) for patients with cancer.

**Design, Setting, and Participants:**

This retrospective, hospital-level, cross-sectional study used longitudinal data from the National Cancer Database from January 1, 2007, to December 31, 2016. Adult patients older than 40 years who had received a diagnosis of 1 of the 10 most common incident cancers and initiated their treatment at a Commission on Cancer–accredited hospital were included. Data were analyzed between December 19, 2019, and March 27, 2020.

**Exposures:**

The mean annual rate of patient volume growth at a hospital.

**Main Outcomes and Measures:**

The main outcome was TTI, defined as the number of days between diagnosis and the first cancer treatment. The association between a hospital’s mean annual rate of patient volume growth and TTI was assessed using a linear mixed-effects model containing a patient volume × time interaction. The mean annual change in TTI over the study period by hospital type was estimated by including a hospital type × time interaction term.

**Results:**

The study sample included 4 218 577 patients (mean [SD] age, 65.0 [11.4] years; 56.6% women) treated at 1351 hospitals. From 2007 to 2016, patient volume increased 40% at NCI centers, 25% at academic centers, and 8% at community hospitals. In 2007, the mean TTI was longer at NCI and academic centers than at community hospitals (NCI: 50 days [95% CI, 48-52 days]; academic: 43 days [95% CI, 42-44 days]; community: 37 days [95% CI, 36-37 days]); however, the mean annual increase in TTI was greater at community hospitals (0.56 days; 95% CI, 0.49-0.62 days) than at NCI centers (−0.73 days; 95% CI, −0.95 to −0.51 days) and academic centers (0.14 days; 95% CI, 0.03-0.26 days). An annual volume growth rate of 100 patients, a level observed at less than 1% of hospitals, was associated with a mean increase in TTI of 0.24 days (95% CI, 0.18-0.29 days).

**Conclusions and Relevance:**

In this cross-sectional study, from 2007 to 2016, across the studied cancer types, patients increasingly initiated their cancer treatment at NCI and academic centers. Although increases in patient volume at these centers outpaced that at community hospitals, faster growth was not associated with clinically meaningful treatment delays.

## Key Points

**Question:**

Is the number of patients with cancer who are treated at referral centers increasing, and are more rapid increases in patient volume associated with treatment delays?

**Findings:**

In this cross-sectional study of 4 218 577 patients treated for an incident cancer at 1351 hospitals from 2007 to 2016, patient volume increased more rapidly at National Cancer Institute and academic centers than at community hospitals, but this was not associated with clinically meaningful increases in time to treatment initiation.

**Meaning:**

Faster patient volume growth at referral centers was not associated with clinically meaningful cancer treatment delays.

## Teaser

This cross-sectional study uses data from the National Cancer Database to investigate trends in patient volume by hospital type and the association of patient volume with time to treatment initiation for patients with cancer by hospital type.

## Introduction

Increasing demand for cancer care services^1,2^ has raised concern of an impending capacity crisis in oncology care.^3,4^ Although an increase in patient volume is expected nationally,^1^ this may be particularly true at referral centers, such as National Cancer Institute (NCI)–designated and academic cancer centers given trends in health care consolidation^5^ and ongoing calls for the regionalization of complex care.^6,7,8,9,10,11^ Although some studies suggest improved outcomes among patients with cancer treated at NCI and academic cancer centers,^12,13,14,15^ increased demand for care at these institutions might exceed their capacity to provide timely cancer treatment^16^ and result in treatment delays.

Population-based studies have suggested that delays in cancer treatment are increasingly common^17,18^ and may occur more frequently at NCI and academic cancer centers.^17,19^ Delays are also disproportionately experienced by individuals from underserved racial/ethnic minority groups^17,20^ and those with low income.^21^ Because delays may be associated with patient distress^22,23^ and worse outcomes,^17,20^ time to treatment initiation (TTI) has been used both as a patient-centered quality metric^24,25^ and as an outcome to evaluate the impact of health policies.^18,26^ However, the mechanisms of recent increases in TTI remain poorly characterized, and to our knowledge, the degree to which hospital capacity strain has contributed to these increases has not been investigated.

Because increases in demand without concurrent increases in capacity may be associated with longer wait times,^27,28,29,30,31^ increases in TTI at centers experiencing greater patient volume growth could provide an early indication of capacity strain.^32,33^ Therefore, the objective of this study was to evaluate hospitals’ patient volume growth rates by hospital type and the association between these growth rates and TTI trends. We hypothesized that patient volume growth would increase more rapidly at NCI and academic centers than at community hospitals and that there would be an association between more rapid patient volume growth and treatment delays.

## Methods

### Study Design

We conducted a retrospective, hospital-level cross-sectional study of longitudinal data from January 1, 2007, to December 31, 2016, to evaluate patient volume growth rates by hospital type. We also evaluated the association between a hospital’s patient volume growth and TTI. The study was deemed exempt from review by the University of Pennsylvania institutional review board; informed consent was not required owing to the use of deidentified registry data. This study followed the Strengthening the Reporting of Observational Studies in Epidemiology (STROBE) reporting guideline.^34^

### Data Source

We used the National Cancer Database (NCDB), a hospital-based registry that captures patient-level data for those who receive either a cancer diagnosis or any part of the first course of treatment at a Commission on Cancer (CoC)–accredited facility. Jointly sponsored by the American College of Surgeons and American Cancer Society, the NCDB includes more than 1500 member institutions, representing approximately one-third of hospitals and nearly three-quarters of annual incident cancer cases in the US.^35,36^ Data from the NCDB are available to investigators upon application to and approval by the CoC.^37^

### Population

The study population included adult patients who received a diagnosis of the following incident cancers from 2007 to 2016 and were treated within 365 days of diagnosis: breast, lung, prostate, colorectal, melanoma, bladder, non-Hodgkin lymphoma, kidney, uterine, or pancreatic. These represent the 10 most commonly diagnosed cancers in the US.^38^ Patients with cancer were identified using the *International Classification of Diseases for Oncology, Third Edition* site and histology codes (eMethods in the Supplement). We excluded individuals with noninvasive or in situ cancers, male breast cancer, small cell lung cancer, noncutaneous melanoma, or nonmuscle invasive bladder cancer. We also excluded patients for whom it was not possible to determine the type of hospital at which they initiated therapy (ie, those initiating treatment at a facility other than the reporting hospital and those who received a diagnosis when they were younger than 40 years, for whom the NCDB suppresses hospital type to prevent hospital identification).

### Measures

The primary outcome was TTI, defined for each patient as the number of days between the date of diagnosis and the start of the first cancer treatment of any type. The TTI was derived from existing NCDB variables describing the number of days between diagnosis and treatment initiation for each of the following modalities: surgery, radiation therapy, and systemic therapy. The exposure was a hospital’s mean annual patient volume growth rate over the study period. Patient volume was defined annually as the total number of patients with an incident cancer diagnosis for whom the reporting hospital initiated a first course of treatment in the specified year. Each hospital was assigned 1 of 4 mutually exclusive designations as codified in the NCDB: community, academic, NCI, or integrated network.^39^

Covariates included age, sex, race/ethnicity (as reported to the NCDB), educational level, income, insurance type, distance from the patient’s residence to the treatment facility, rurality, Charlson-Deyo comorbidity score,^40^ history of cancer, cancer type, cancer stage, treatment modality, and whether care was transferred between the diagnosis and the start of treatment.

### Statistical Analysis

We evaluated the association between a hospital’s mean annual patient volume growth rate and TTI using a linear mixed-effects model containing a patient volume × time interaction. The mean annual change in TTI over the study period by hospital type was estimated by including a hospital type × time interaction term. To estimate the mean annual patient volume growth rate by hospital type, we separately modeled patient volume as an outcome. Additional details of the model specifications are given in the eMethods in the Supplement. Our linear mixed-effects models contained a random time slope and a random intercept for each individual hospital (as identified by their facility number) to account for their differing growth rates. A hospital’s unique facility number was retained over time except in the case of hospital mergers or acquisitions, after which the consolidated entity adopted a new, distinct identification number. This approach allowed us to account for confounding owing to hospital mergers that would have otherwise produced large absolute increases in volume during the study period by essentially modeling separate entities before and after the merger or acquisition. We conducted secondary stratified analyses to examine trends by cancer type and initial treatment modality. Missing covariate data were imputed using multiple imputation by chained equations.^41^ The mean of 5 imputed data sets was calculated, and SEs were estimated using the Rubin formula.^42^

We were concerned a priori that increases in active surveillance of prostate cancer during the study period^43,44^ might result in a greater number of patients with low-risk prostate cancer managed with active surveillance rather than treatment initiation over time. Because the study population consisted of patients undergoing a first course of treatment, such changes could affect the total number of patients with prostate cancer included in the sample as well as the proportion of those patients with aggressive disease. Therefore, we designed a principal sensitivity analysis excluding patients with prostate cancer to test whether their inclusion biased our results. We also conducted the following additional sensitivity analyses to test key variable definitions and model specifications: an extreme case sensitivity analysis excluding patients with a TTI of 0, an analysis including only patients treated within 180 days of diagnosis, an analysis including only the 85% of hospitals continuously represented in the data set during all 10 years of our study (because hospitals may have gained or lost CoC accreditation), and an analysis including cases in which treatment was initiated outside the reporting facility. Although TTI for patients treated at another hospital was unlikely to be determined by the reporting hospital, such patients still contributed to annual patient volume, and therefore, their inclusion could have potentially altered the association between the mean annual patient volume growth and TTI.

Data were analyzed using Stata software, version 15 (StataCorp LLC) and RStudio, version 1.1.463 (RStudio Team) between December 19, 2019, and March 27, 2020. We evaluated our hypotheses using 2-tailed tests at a significance level of *P* = .02 (Bonferroni correction for testing 3 primary hypotheses^45^).

## Results

### Study Population

We identified 4 218 577 patients (mean [SD] age, 65.0 [11.4] years; 56.6% women) who received a diagnosis of an incident cancer from 2007 to 2016 and initiated a first course of treatment at 1351 hospitals (49.3% at 897 community hospitals, 23.2% at 177 academic hospitals, 13.8% at 50 NCI hospitals, and 13.7% at 227 integrated network hospitals). The study population flow diagram is shown in the eFigure in the Supplement. Table 1 presents patient demographic and clinical characteristics by hospital type. Compared with patients treated at community hospitals, those treated at academic and NCI centers more frequently were male, were non-White individuals, had higher income, had a higher educational level, were privately insured, were residents of urban areas, had a diagnosis of stage IV disease, received systemic therapy, and had care transferred to the treating hospital.

**Table 1.** Patient Demographic and Clinical Characteristics by Hospital Type From 2007 to 2016

Variable	Patients^a^				
	Community hospital (n = 2 078 051)	Academic hospital (n = 979 318)	NCI hospital (n = 583 994)	Integrated hospital (n = 577 214)	Total (N = 4 218 577)
Age, mean (SD), y	65.8 (11.5)	64.2 (11.2)	63.3 (10.9)	65.0 (11.4)	65.0 (11.4)
Sex					
Male	865 016 (41.6)	430 780 (44.0)	294 081 (50.4)	241 124 (41.8)	1 831 001 (43.4)
Female	1 213 035 (58.4)	548 538 (56.0)	289 913 (49.6)	336 090 (58.2)	2 387 576 (56.6)
Race					
White	1 818 997 (87.5)	774 035 (79.0)	487 788 (83.5)	487 886 (84.5)	3 568 706 (84.6)
Black	186 504 (9.0)	149 189 (15.2)	63 963 (11.0)	66 508 (11.5)	466 164 (11.1)
Asian	43 967 (2.1)	32 357 (3.3)	17 137 (2.9)	10 589 (1.8)	104 050 (2.5)
American Indian	7367 (0.4)	1612 (0.2)	1895 (0.3)	833 (0.1)	11 707 (0.3)
Other	9496 (0.5)	10 720 (1.1)	5195 (0.9)	4458 (0.8)	29 869 (0.7)
Missing	11 720 (0.6)	11 405 (1.2)	8016 (1.4)	6940 (1.2)	38 081 (0.9)
Ethnicity					
Non-Hispanic	1 902 354 (91.5)	869 020 (88.7)	543 230 (93.0)	522 444 (90.5)	3 837 048 (91.0)
Hispanic	77 450 (3.7)	59 226 (6.0)	25 360 (4.3)	24 467 (4.2)	186 503 (4.4)
Missing	98 247 (4.7)	51 072 (5.2)	15 404 (2.6)	30 303 (5.2)	195 026 (4.6)
Income^b^					
Quartile 1	361 532 (17.4)	180 533 (18.4)	85 470 (14.6)	82 909 (14.4)	710 444 (16.8)
Quartile 2	508 735 (24.5)	183 914 (18.8)	116 274 (19.9)	119 498 (20.7)	928 421 (22.0)
Quartile 3	543 412 (26.2)	232 049 (23.7)	133 417 (22.8)	156 536 (27.1)	1 065 414 (25.3)
Quartile 4	658 604 (31.7)	380 273 (38.8)	246 984 (42.3)	216 514 (37.5)	1 502 375 (35.6)
Missing	5768 (0.3)	2549 (0.3)	1849 (0.3)	1757 (0.3)	11 923 (0.3)
Educational level^c^					
Quartile 1	357 873 (17.2)	193 626 (19.8)	86 261 (14.8)	74 793 (13.0)	712 553 (16.9)
Quartile 2	546 606 (26.3)	242 737 (24.8)	130 183 (22.3)	138 133 (23.9)	1 057 659 (25.1)
Quartile 3	673 007 (32.4)	278 269 (28.4)	176 353 (30.2)	196 961 (34.1)	1 324 590 (31.4)
Quartile 4	495 960 (23.9)	262 555 (26.8)	189 629 (32.5)	165 874 (28.7)	1 114 018 (26.4)
Missing	4605 (0.2)	2131 (0.2)	1568 (0.3)	1453 (0.3)	9757 (0.2)
Insurance					
Uninsured	43 181 (2.1)	37 407 (3.8)	10 410 (1.8)	10 615 (1.8)	101 613 (2.4)
Private	846 780 (40.7)	425 105 (43.4)	273 302 (46.8)	248 678 (43.1)	1 793 865 (42.5)
Medicaid	88 692 (4.3)	66 032 (6.7)	27 641 (4.7)	27 025 (4.7)	209 390 (5.0)
Medicare	1 050 978 (50.6)	424 020 (43.3)	235 265 (40.3)	279 725 (48.5)	1 989 988 (47.2)
Other government	25 139 (1.2)	10 919 (1.1)	9060 (1.6)	6378 (1.1)	51 496 (1.2)
Missing	23 281 (1.1)	15 835 (1.6)	28 316 (4.8)	4793 (0.8)	72 225 (1.7)
Charlson-Deyo comorbidity score					
0	1 502 788 (72.3)	733 013 (74.8)	453 972 (77.7)	418 045 (72.4)	3 107 818 (73.7)
1	417 813 (20.1)	179 081 (18.3)	96 595 (16.5)	114 434 (19.8)	807 923 (19.2)
2	111 789 (5.4)	46 015 (4.7)	23 062 (3.9)	31 180 (5.4)	212 046 (5.0)
≥3	45 661 (2.2)	21 209 (2.2)	10 365 (1.8)	13 555 (2.3)	90 790 (2.2)
Prior cancer					
No	1 723 818 (83.0)	816 725 (83.4)	474 573 (81.3)	476 094 (82.5)	3 491 210 (82.8)
Yes	354 135 (17.0)	162 542 (16.6)	109 409 (18.7)	101 038 (17.5)	727 124 (17.2)
Missing	98 (0.0)	51 (0.0)	12 (0.0)	82 (0.0)	243 (0.0)
Distance to hospital, median (IQR), mi	8.9 (4.0-19.7)	10.0 (4.6-23.4)	23.7 (9.2-62.9)	8.6 (4.2-17.1)	10.1 (4.5-23.6)
Transfer of care					
No	1 511 776 (72.7)	627 456 (64.1)	243 245 (41.7)	393 567 (68.2)	2 776 044 (65.8)
Yes	566 275 (27.3)	351 862 (35.9)	340 749 (58.3)	183 647 (31.8)	1 442 533 (34.2)
Geographic location					
Metropolitan	1 617 679 (77.8)	856 553 (87.5)	483 607 (82.8)	505 543 (87.6)	3 463 382 (82.1)
Nonmetropolitan	410 826 (19.8)	102 638 (10.5)	86 922 (14.9)	48 841 (8.5)	649 227 (15.4)
Missing	49 546 (2.4)	20 127 (2.1)	13 465 (2.3)	22 830 (4.0)	105 968 (2.5)
Cancer type					
Breast	654 927 (31.5)	252 221 (25.8)	119 379 (20.4)	169 845 (29.4)	1 196 372 (28.4)
Lung	319 384 (15.4)	148 390 (15.2)	89 665 (15.4)	84 292 (14.6)	641 731 (15.2)
Prostate	344 016 (16.6)	181 829 (18.6)	127 272 (21.8)	101 910 (17.7)	755 027 (17.9)
Colon	270 978 (13.0)	92 056 (9.4)	32 755 (5.6)	68 508 (11.9)	464 297 (11.0)
Rectal	48 204 (2.3)	21 506 (2.2)	13 025 (2.2)	11 691 (2.0)	94 426 (2.2)
Melanoma	64 361 (3.1)	38 973 (4.0)	40 446 (6.9)	20 477 (3.5)	164 257 (3.9)
Bladder	22 944 (1.1)	12 578 (1.3)	10 631 (1.8)	5939 (1.0)	52 092 (1.2)
NHL^d^	85 043 (4.1)	44 027 (4.5)	36 221 (6.2)	20 871 (3.6)	186 162 (4.4)
Kidney	115 368 (5.6)	67 353 (6.9)	45 456 (7.8)	36 390 (6.3)	264 567 (6.3)
Uterine	111 300 (5.4)	87 429 (8.9)	38 329 (6.6)	43 487 (7.5)	280 545 (6.7)
Pancreatic	41 526 (2.0)	32 956 (3.4)	30 815 (5.3)	13 804 (2.4)	119 101 (2.8)
Cancer stage					
I	847 390 (40.8)	401 617 (41.0)	218 190 (37.4)	246 066 (42.6)	1 713 263 (40.6)
II	656 528 (31.6)	301 344 (30.8)	179 625 (30.8)	182 014 (31.5)	1 319 511 (31.3)
III	337 202 (16.2)	158 073 (16.1)	98 817 (16.9)	90 929 (15.8)	685 021 (16.2)
IV	236 931 (11.4)	118 284 (12.1)	87 362 (15.0)	58 205 (10.1)	500 782 (11.9)
First treatment					
Surgery	1 497 871 (72.1)	692 898 (70.8)	387 950 (66.4)	437 582 (75.8)	3 016 301 (71.5)
Radiation	230 853 (11.1)	103 854 (10.6)	50 130 (8.6)	62 649 (10.9)	447 486 (10.6)
Systemic	349 327 (16.8)	182 566 (18.6)	145 914 (25.0)	76 983 (13.3)	754 790 (17.9)

Abbreviations: IQR, interquartile range; NCI, National Cancer Institute; NHL, non-Hodgkin lymphoma.

^a^ Data are presented as number (percentage) of patients unless otherwise specified.

^b^ Quartile at the zip code level. From 2007 to 2012, quartile 1 was less than $38 000; quartile 2, $38 000 to $47 999; quartile 3, $48 000 to $62 999; and quartile 4, greater than $63 000. From 2013 to 2016, quartile 1 was less than $40 227; quartile 2, $40 227 to $50 353; quartile 3, $50 354 to $63 332; and quartile 4, greater than $63 333.

^c^ Quartiles at the zip code level for the percentage of adults who did not graduate high school. From 2007 to 2012, quartile 1 was 21% or more; quartile 2, 13% to 20.9%; quartile 3, 7% to 12.9%; and quartile 4, less than 7%. From 2013 to 2016, quartile 1 was 17.6% or more; quartile 2, 10.9% to 17.5%; quartile 3, 6.3% to 10.8%; and quartile 4, less than 6.3%.

^d^ Includes chronic lymphocytic leukemia and small lymphocytic lymphoma.

### Patient Volume Growth by Hospital Type

**Figure 1.**
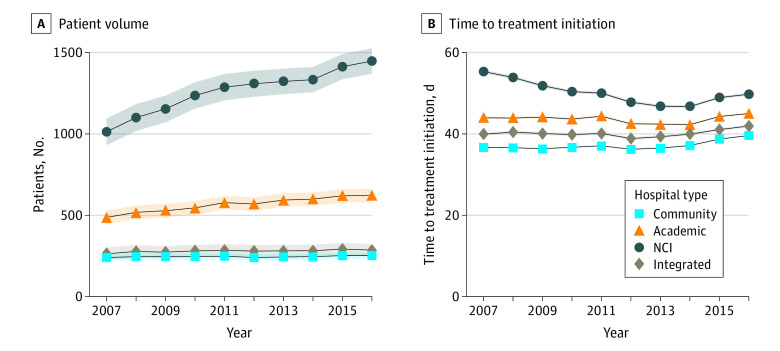
Trends in Patient Volume and Time to Treatment Initiation Among All Cancer Types by Hospital Type From 2007 to 2016 Shaded areas represent 95% CIs. NCI indicates National Cancer Institute.

**Figure 2.**
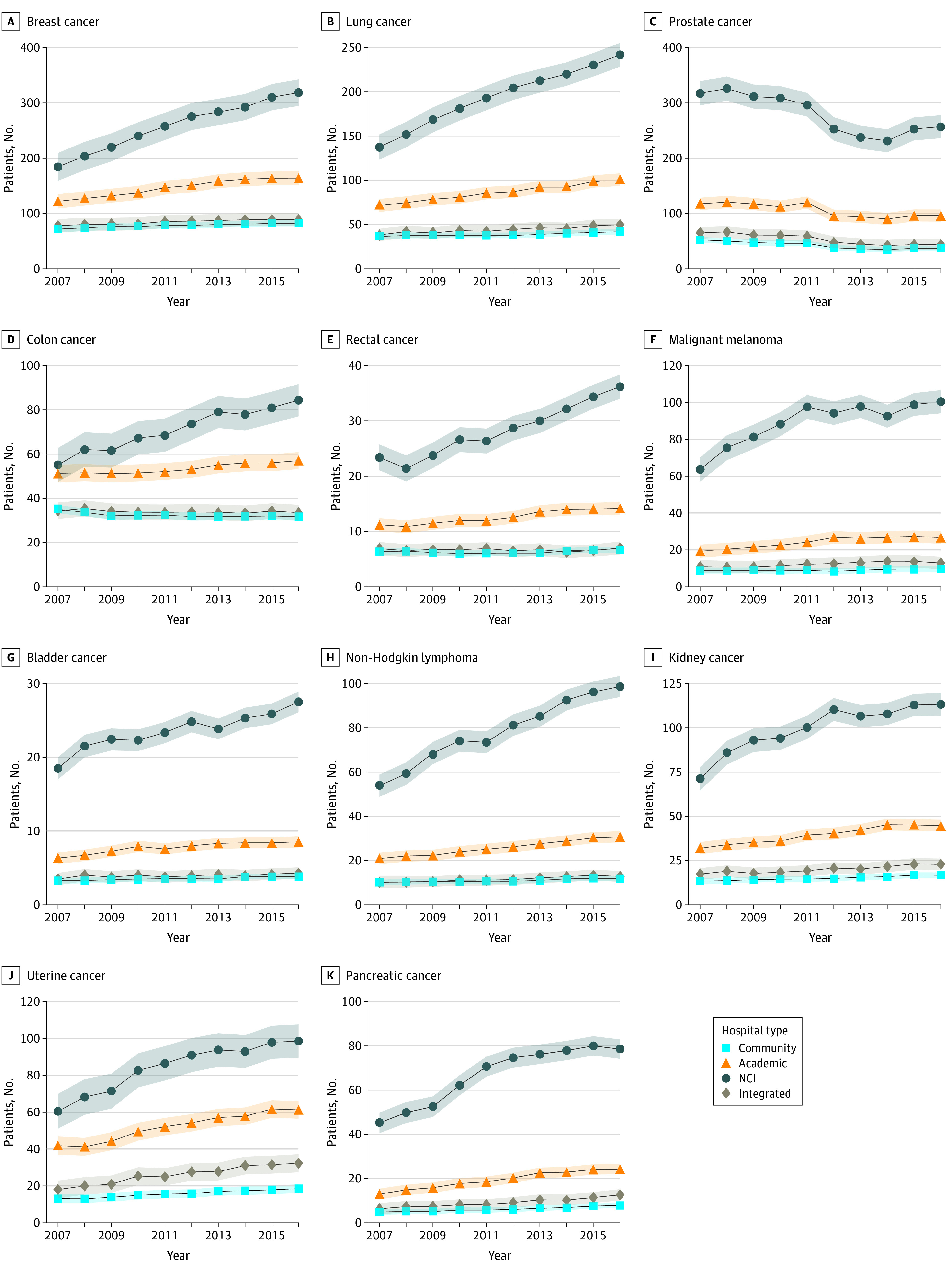
Trends in Patient Volume by Hospital Type and Cancer Type From 2007 to 2016 Shaded areas represent 95% CIs. NCI indicates National Cancer Institute.

In 2007, the mean number of patients with incident cancer diagnoses who had treatment initiated at NCI hospitals was 1027 (95% CI, 960-1093 patients), at academic hospitals was 505 (95% CI, 470-540 patients), and at community hospitals was 232 (95% CI, 217-248 patients) (Table 2). Figure 1A shows trends in patient volume by hospital type. From 2007 to 2016, patient volume increased by 40% at NCI hospitals, 25% at academic hospitals, and 8% at community hospitals; the mean annual patient volume growth rate was 45.2 patients (95% CI, 38.1-52.2 patients) at NCI hospitals and 13.9 patients (95% CI, 10.2-17.5 patients) at academic hospitals compared with 2.0 patients (95% CI, 0.4-3.7 patients) at community hospitals (Table 2). In analyses stratified by cancer type, NCI and academic hospitals experienced significantly higher mean annual patient volume growth compared with community hospitals across all cancer types except prostate cancer (Figure 2 and eTable in the Supplement). Growth rates were proportionally greatest at NCI centers for patients with non-Hodgkin lymphoma and pancreatic cancer; for both cancer types, the volume increased 69% over the study period. Increases in volume were lower for bladder (30%) and kidney (38%) cancers. When stratified by treatment modality, growth rates were higher at NCI and academic centers regardless of whether patients were initially treated with surgery or systemic therapy.

**Table 2.** Level and Annual Growth Rate of Patient Volume and Adjusted TTI by Hospital Type, 2007-2016

Variable	Community^a^	Academic	NCI	Integrated
Patients treated, mean (95% CI), No.				
2007	232 (217 to 248)	505 (470 to 540)^b^	1027 (960 to 1093)^b^	248 (217 to 279)
2016	251 (229 to 272)	630 (582 to 678)^b^	1433 (1342 to 1524)^b^	278 (235 to 321)
Mean annual patient volume growth rate, mean (95% CI), No.	2.0 (0.4 to 3.7)	13.9 (10.2 to 17.5)^b^	45.2 (38.1 to 52.2)^b^	3.3 (0.0 to 6.6)
Adjusted TTI, mean (95% CI), d				
2007	37 (36 to 37)	43 (42 to 44)^b^	50 (48 to 52)^b^	38 (37 to 39)
2016	42 (41 to 42)	44 (43 to 45)^c^	43 (42 to 45)	42 (41 to 43)
Mean annual TTI growth rate, mean (95% CI), d	0.56 (0.49 to 0.62)	0.14 (0.03 to 0.26)^b^	−0.73 (−0.95 to −0.51)^b^	0.51 (0.39 to 0.63)

Abbreviations: NCI, National Cancer Institute; TTI, time to treatment initiation.

^a^ Reference.

^b^ *P* < .001 compared with reference.

^c^ *P* = .003.

### Trends in TTI by Hospital Type

**Figure 3.**
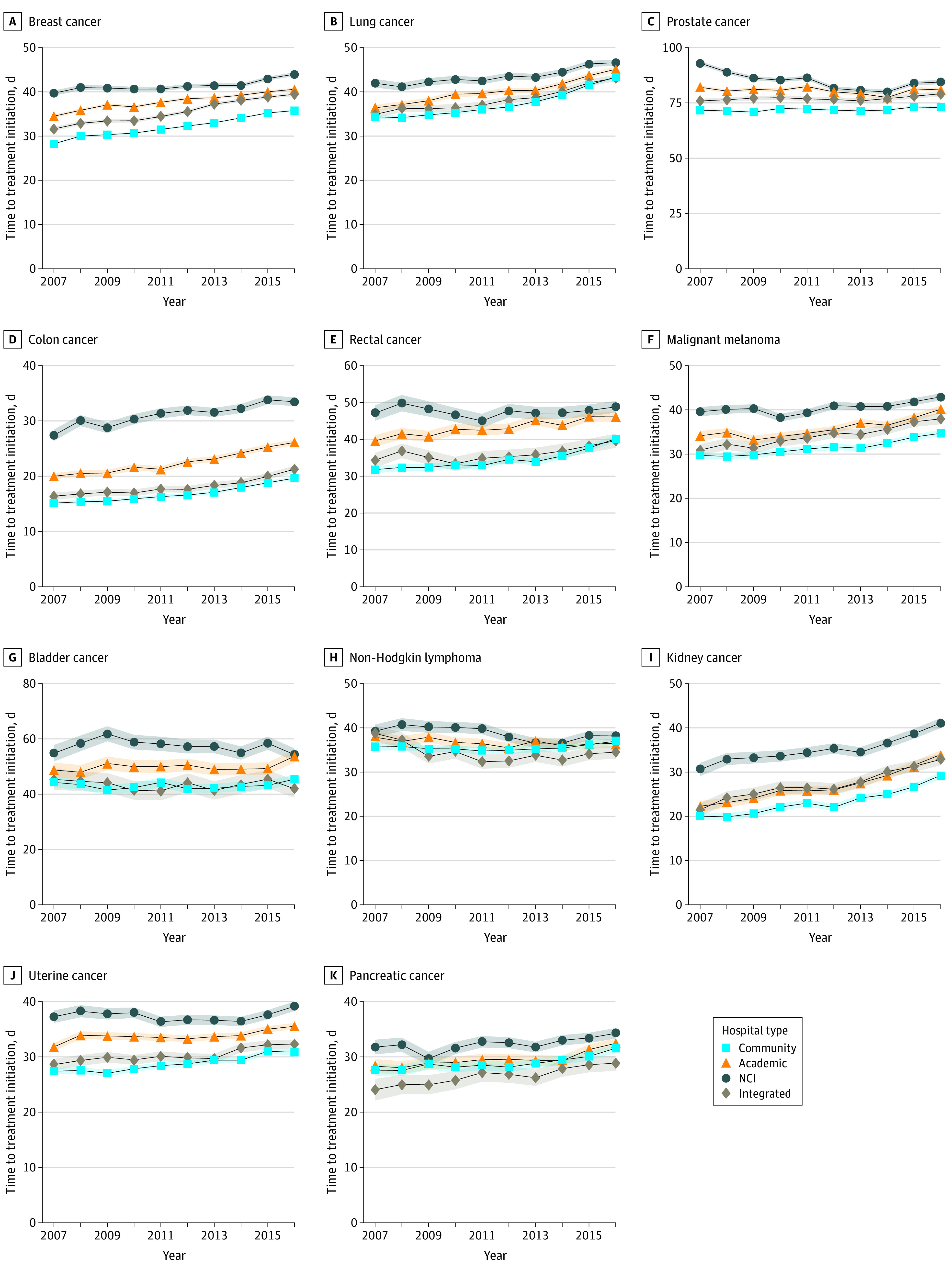
Trends in Unadjusted Time to Treatment Initiation by Hospital Type and Cancer Type From 2007 to 2016 Shaded areas represent 95% CIs. NCI indicates National Cancer Institute.

Figure 1B shows trends in TTI by hospital type before adjustment for patient, tumor, and treatment factors. Figure 3 shows unadjusted TTI trends stratified by cancer type. Unadjusted TTI was longer at NCI and academic hospitals than at community hospitals throughout the study period (Figure 1B). The adjusted TTI in 2007 and 2016 by hospital type and the mean annual TTI growth rates by hospital type are shown in Table 2. After adjustment, in 2007, the mean TTI was significantly longer at NCI (50 days; 95% CI, 48-52 days) and academic (43 days; 95% CI, 42-44 days) hospitals than at community hospitals (37 days; 95% CI, 36-37 days). However, the adjusted annual mean TTI growth rate was greater at community hospitals (0.56 days; 95% CI, 0.49-0.62 days) than at NCI centers (−0.73 days; 95% CI, −0.95 to −0.51 days), where there was a mean annual reduction in TTI, and academic hospitals (0.14 days; 95% CI, 0.03-0.26 days). Therefore, by 2016, the difference in the adjusted TTI between academic and community hospitals had attenuated (44 days [95% CI, 43 to 45 days] vs 42 days [95% CI, 41 to 42 days]), and there was no statistically significant difference in the mean adjusted TTI between NCI hospitals (43 days; 95% CI, 42-45 days) and community hospitals (42 days; 95% CI, 41-42 days) (Table 2).

### Association of Patient Volume Growth Rate With Changes in TTI and Results of Sensitivity Analyses

Mean annual volume growth of 100 patients, a level observed in less than 1% of hospitals in the sample, was associated with an annual mean increase in TTI of 0.24 days (95% CI, 0.18-0.29 days). The association between this level of annual patient volume growth and changes in TTI was similar across hospital types (NCI centers: 0.55 days [95% CI, 0.09 to 1.01 days]; academic hospitals: −0.04 days [95% CI, −0.72 to 0.64 days]; community hospitals: 0.17 days [95% CI, −0.38 to 0.72 days]). The results of models stratified by cancer type did not demonstrate an association between greater volume growth and increases in TTI. The results of our sensitivity analyses excluding prostate cancer, excluding patients with a TTI of 0, including only patients treated within 180 days of diagnosis, including only continuously represented hospitals, and including cases in which treatment was initiated outside the reporting facility were similar to those of the primary analysis.

## Discussion

From 2007 to 2016, NCI and academic centers experienced greater patient volume growth than did community hospitals for all but 1 of the 10 cancer types included in our study. However, over the same period, TTI increased most rapidly at community hospitals, while the mean TTI decreased over time at NCI centers. We found no evidence that receipt of treatment at hospitals with greater patient volume growth was associated with clinically meaningful treatment delays. Our study is, to our knowledge, the first to investigate whether rapid patient volume growth is associated with capacity constraints leading to cancer treatment delays. The findings suggest that NCI and academic centers had or developed sufficient capacity during this period to treat an increasing number of patients with newly diagnosed cancer without incurring delays in treatment.

Increases in the demand for cancer care in the US have not been evenly distributed, with NCI and academic centers treating an increasing share of the country’s incident cancer cases across cancer types. Greater increases in patient volume at referral centers may reflect increasing health care consolidation over the past decade^5^ and an increasing tendency for community hospitals that are affiliated with NCI and academic centers to refer patients centrally for oncologic care. Although there is some evidence that health systems–based centralization of cancer care has occurred,^46^ it remains unclear whether acquisitions and affiliations are associated with improvement in either care processes or patient outcomes.^47,48^ Financial incentives may also contribute to patient volume growth given that high revenues in oncology^49^ have been shown to incentivize hospitals to expand their cancer treatment capacity.^50^ Also, increased volume at referral centers might be the result of specific efforts aimed at regionalizing cancer care, but these efforts have focused primarily on specific complex cancer surgeries, such as pancreatic, esophageal, lung, and rectal cancer resections,^51,52^ and our analysis demonstrated higher patient volume growth rates at NCI and academic hospitals across cancer types and for both surgical and systemic therapy.

In contrast to our hypothesis, we observed the greatest increase in TTI among community hospitals, where the slowest increases in patient volume occurred, whereas TTI decreased at NCI centers despite higher mean annual volume growth. The lack of association between the increasing number of patients treated at referral centers and treatment delays might reflect efficiency gains, expanded capacity, or quality improvement efforts during the study period. Previous efforts have shown that hospitals can increase their efficiency through interventions to improve communication, enhance coordination, and leverage technology.^53^

Although referral centers appear to have met the increased demand for cancer care without delaying treatment, our results suggest several areas for improvement. First, patients treated at NCI and academic centers waited longer to start treatment than did patients at community hospitals before adjustment for patient population. However, when adjusting for case mix and facility transfer, this difference was eliminated or attenuated, respectively, in later years. This may reflect a number of factors, such as a need to gather multidisciplinary input into treatment decisions, to reevaluate data that were obtained at another site, or to repeat tissue sample obtainment.^54^ Second, for most of the cancers studied, TTI increased regardless of hospital type. Although this trend may partially reflect the increasing complexity of treatment decisions and a desire to consider the results of molecular testing when making frontline treatment decisions, previous work has shown that hospitals can successfully implement programs that reduce treatment delays.^53^ Because of the potential association of such delays with psychosocial distress^22,23^ and survival,^17,20^ continued efforts to ensure timely cancer treatment are warranted.

### Limitations

This study has limitations. First, this was an observational study, and although our linear mixed-effects model accounted for both observed changes in hospital case mix over time and hospital-specific factors, we could not rule out bias owing to unobserved changes in a hospital’s patient population over time. We performed several sensitivity analyses to test the robustness of our findings to potentially time-varying characteristics, and each yielded results that were similar to those of the primary analysis. A second limitation was that the NCDB is a hospital-based registry, and inclusion in the database depends on CoC accreditation. Hospitals without CoC accreditation may have had trends that differed from those we observed. However, because our hypotheses focused on changes over time at the hospital level, a hospital-based registry was well suited for the present analysis. Furthermore, the NCDB contains nearly three-quarters of all incident cancer diagnoses in the US and is likely to reflect the circumstances in which most patients receive their cancer care. In addition, although the findings of our study are representative of only the cancer types that we selected and that were treated in the first-line setting during the study period, these cancer types collectively represented more than half of incident cancer diagnoses in the US. Future studies should seek to understand whether the results are generalizable to patients with other cancer types and to those seeking second- or later-line therapy. Further investigation is also needed to understand whether referral centers will continue to be able to expand their capacity at the rates observed in our study.

## Conclusions

In this cross-sectional study, patient volume growth was most rapid at NCI and academic centers across cancer types, but more rapid growth was not associated with clinically meaningful delays in cancer treatment during the study period. Additional investigation is needed to determine whether the observed patient volume growth rates are sustainable.

## Notes

Supplement. **eMethods.** ICD-O-3 Site and Histology Codes Used to Define Cancer Types **eFigure.** Patient Flow Diagram **eTable.** Level and Annual Growth Rate of Patient Volume by Cancer Type
