# Supplementary material for: Trends in Patient Volume by Hospital Type and the Association of These Trends With Time to Cancer Treatment Initiation
Source: JAMA Netw Open. 2021 Jul 9;4(7):e2115675. doi: 10.1001/jamanetworkopen.2021.15675 (PMC8271360; doi:10.1001/jamanetworkopen.2021.15675)
Supplement: Supplement. — eMethods. ICD-O-3 Site and Histology Codes Used to Define Cancer Types eFigure. Patient Flow Diagram eTable. Level and Annual Growth Rate of Patient Volume by Cancer Type [file jamanetwopen-e2115675-s001.pdf]

Supplementary Online Content

Frosch ZAK, Illenberger N, Mitra N, et al. Trends in patient volume by hospital type and the association of these trends with time to cancer treatment initiation. *JAMA Netw Open*. 2021;4(7):e2115675. doi:10.1001/jamanetworkopen.2021.15675

eMethods. ICD-O-3 Site and Histology Codes Used to Define Cancer Types

eFigure. Patient Flow Diagram

eTable. Level and Annual Growth Rate of Patient Volume by Cancer Type

This supplementary material has been provided by the authors to give readers additional information about their work.

© 2021 Frosch ZAK et al. *JAMA Network Open.*

eMethods. ICD-O-3 Site and Histology Codes Used to Define Cancer Types

Breast: C50.0-50.9

Non-small cell lung: C34.0-34.9 excluding histology codes 8041-8045

Prostate: C61.9

Colon: C18.0-18.9

Rectum: C20.9

Melanoma: C44.0-44.9 with histology codes 8720-23, 8728, 8730, 8740-46, 8761, 8770-74 or 8780

Bladder: C67.0-67.9

NHL: histology codes 9590-9597, 9670-9699, 9700-9729, 9735-9738, 9761, and 9811-9837

Renal: C64.9 or C65.9

Uterus: C54.0-54.9

Pancreas: C25.0-25.9

Model specifications and hypothesis testing

Each of our linear mixed-effects model allowed for differing growth rates between individual hospitals (random time slope) as well as differing means (random intercept). Variable inclusion was based on purposeful selection a priori. The models were:

TTI model:

$$TTI_{ijt} = \beta_0 + \beta_1 T + \beta_2 PV_{jt} + \beta_3 hosp\_type + \beta_4 (T \text{ x } hosp\_type) + \beta_5 (T \text{ x } PV_{jt}) + \beta_6 X_{ijt} + \alpha_{0j} + \alpha_{1j} T + e_{ijt}$$

Where i denotes the individual patient, j denotes the hospital and t denotes the year of diagnosis.

© 2021 Frosch ZAK et al. *JAMA Network Open.*

PV_{jt} is the annual patient volume for the hospital at which the patient was treated in the year that the patient was diagnosed and X_{ijt} is a vector of individual characteristics: age, sex, race, ethnicity, education and income (at the zip-code level based on the American Community Survey), insurance type, distance from the patient's residence to the treating facility, rurality (metro vs non-metro by rural-urban continuum codes), Charlson-Deyo comorbidity score (1, 2 or 3+), history of prior malignancies, cancer type, stage, treatment modality, and whether care was transferred between diagnosis and the start of treatment. T is the year of a patient's diagnosis, $\alpha_{1j}T$ denotes the random hospital time slope, and α_{0j} the random hospital intercept.

To examine the relationship between patient volume growth and TTI by hospital type, we conducted analyses stratified by hospital type. In these models, the variables hosp_type and T x hosp_type were not included, but the model was otherwise identical to the above.

Patient volume model:

$$PV_{jt} = \beta_0 + \beta_1 T + \beta_2 hosp\_type + \beta_3 (T \times hosp\_type) + \alpha_{0j} + \alpha_{1j}T + e_{jt}$$

Where j denotes the hospital and t denotes the year of diagnosis. Again, PV_{jt} is the annual patient volume in that year, T is the year of a patient's diagnosis, $\alpha_{1j}T$ denotes the random hospital time slope, and α_{0j} the random hospital intercept.

Community hospitals were the reference group for hospital type in both models. We tested for an association between volume growth and TTI using a Wald test of a linear combination of β_2 and β_5, which represents the association between patient volume growth and TTI while accounting

© 2021 Frosch ZAK et al. *JAMA Network Open.*

for changes in this association over time. β_4 is the other parameter of interest in the TTI model. β_3 was the parameter of interest in the patient volume model. We tested for differences in growth rates between hospital types using the Wald test on the coefficients of our hospital type-by-time interaction terms (β_4 for TTI and β_3 for patient volume).

© 2021 Frosch ZAK et al. *JAMA Network Open.*

eFigure. Patient Flow Diagram

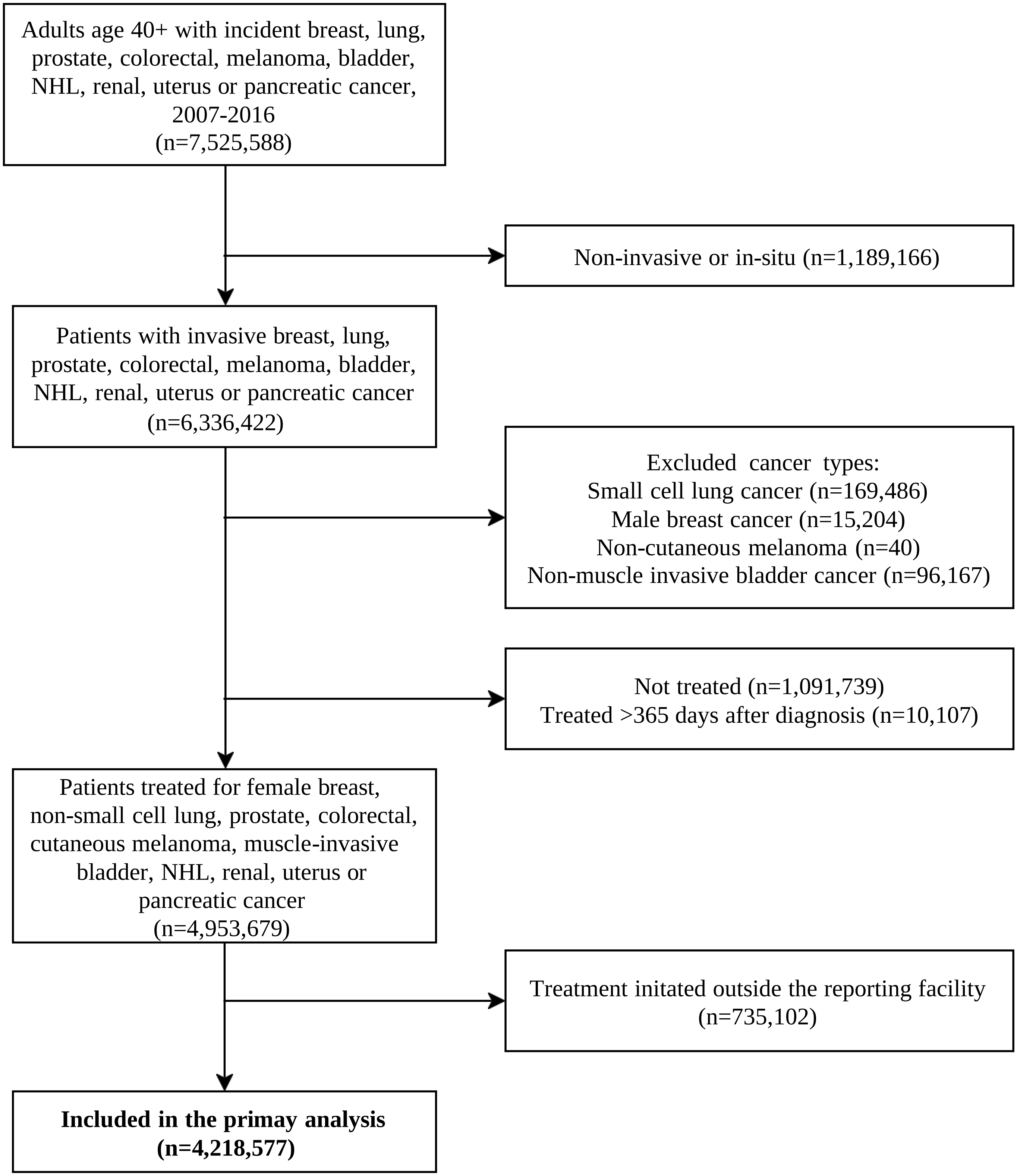

© 2021 Frosch ZAK et al. *JAMA Network Open.*

eTable. Level and Annual Growth Rate of Patient Volume by Cancer Type

	Community[a]	Academic	NCI	Integrated
**Breast**				
Mean no. patients treated (95% CI), 2007	71<br>(66, 75)	124[c]<br>(113, 134)	200[c]<br>(180, 220)	72<br>(63, 81)
Mean no. patients treated (95% CI), 2016	83<br>(77, 90)	169[c]<br>(155, 184)	314[c]<br>(286, 341)	86<br>(73.05, 99.02)
Average annual growth rate, 2007-16<br>- patients (95% CI)	1.4<br>(1.0, 1.8)	5.1[c]<br>(4.2, 6.0)	13[c]<br>(11, 14)	1.6<br>(0.7, 2.4)
**Lung**				
Mean no. patients treated (95% CI), 2007	34<br>(32, 37)	71[c]<br>(66, 77)	151[c]<br>(140, 162)	35<br>(30, 40)
Mean no. patients treated (95% CI), 2016	40<br>(37, 44)	101[c]<br>(92, 109)	237[c]<br>(221, 253)	45<br>(37, 52)
Average annual growth rate, 2007-16<br>- patients (95% CI)	0.7<br>(0.4, 0.9)	3.3[c]<br>(2.7, 3.8)	9.5[c]<br>(8.4, 10.7)	1.1<br>(0.6, 1.6)
**Prostate**				
Mean no. patients treated (95% CI), 2007	48<br>(43, 54)	124[c]<br>(111, 137)	312[c]<br>(287, 336)	59<br>(47, 70)
Mean no. patients treated (95% CI), 2016	32<br>(28, 36)	89[c]<br>(80, 98)	226[c]<br>(210, 243)	37<br>(29, 45)
Average annual growth rate, 2007-16<br>- patients (95% CI)	-1.8<br>(-2.3, -1.3)	-3.8[c]<br>(-4.9, -2.8)	-9.5[c]<br>(-11.5, -7.4)	-2.4<br>(-3.4, -1.5)
**Colon**				
Mean no. patients treated (95% CI), 2007	32<br>(31, 34)	50[c]<br>(47, 54)	59[c]<br>(52, 66)	32<br>(29, 35)
Mean no. patients treated (95% CI), 2016	31<br>(29, 33)	57[c]<br>(53, 61)	83[c]<br>(75, 90)	33<br>(29, 36)
Average annual growth rate, 2007-16<br>- patients (95% CI)	-0.1<br>(-0.3, -0.0)	0.7[c]<br>(0.5, 1.0)	2.6[c]<br>(2.1, 3.1)	0.1<br>(-0.2, 03)
**Rectal**				
Mean no. patients treated (95% CI), 2007	5.8<br>(5.4, 6.2)	11[c]<br>(10, 12)	23[c]<br>(21, 24)	6.0<br>(5.2, 6.8)
Mean no. patients treated (95% CI), 2016	6.0<br>(5.5, 6.6)	14[c]<br>(13, 16)	35[c]<br>(32, 37)	5.9<br>(4.7, 7.1)
Average annual growth rate, 2007-16<br>- patients (95% CI)	0.02<br>(-0.03, 0.08)	0.4[c]<br>(0.3, 0.5)	1.4[c]<br>(1.1, 1.6)	-0.01<br>(-0.13, 0.10)
**Melanoma**				
Mean no. patients treated (95% CI), 2007	7.6<br>(6.3, 9.0)	19[c]<br>(16, 22)	71[c]<br>(66, 77)	9.4<br>(6.7, 12.1)
Mean no. patients treated (95% CI), 2016	8.3<br>(6.7, 9.9)	27[c]<br>(23, 30)	102[c]<br>(95, 109)	12<br>(8, 15)
Average annual growth rate, 2007-16<br>- patients (95% CI)	0.08<br>(-0.10, 0.26)	0.8[c]<br>(0.4, 1.2)	3.4[c]<br>(2.7, 4.2)	0.2<br>(-0.1, 0.6)
**Bladder**				
Mean no. patients treated (95% CI), 2007	3.0<br>(2.7, 3.3)	6.3[c]<br>(5.8, 6.9)	20[c]<br>(19, 21)	3.2<br>(2.6, 3.7)
Mean no. patients treated (95% CI), 2016	3.4<br>(3.1, 3.8)	8.5[c]<br>(7.8, 9.3)	26[c]<br>(25, 28)	3.6<br>(2.9, 4.3)
Average annual growth rate, 2007-16<br>- patients (95% CI)	0.05<br>(0.01, 0.08)	0.2[c]<br>(0.2, 0.3)	0.8<br>(0.6, 0.9)	0.05<br>(-0.02, 0.12)
**NHL[b]**				
Mean no. patients treated (95% CI), 2007	9.4	21[c]	58[c]	8.6

© 2021 Frosch ZAK et al. *JAMA Network Open.*

	(8.6, 10.3)	(19, 22)	(54, 61)	(6.9, 10.4)
Mean no. patients treated (95% CI), 2016	11 (10, 13)	31[c] (28, 34)	98[c] (92, 104)	12 (9, 15)
Average annual growth rate, 2007-16 - patients (95% CI)	0.2 (0.1, 0.3)	1.1[c] (0.9, 1.4)	4.5[c] (4.0, 4.9)	0.4 (0.1, 0.6)
**Renal**				
Mean no. patients treated (95% CI), 2007	12 (11, 14)	32[c] (29, 35)	82[c] (76, 87)	15 (13, 18)
Mean no. patients treated (95% CI), 2016	16 (14, 17)	46[c] (42, 50)	113[c] (107, 120)	20[d] (17, 24)
Average annual growth rate, 2007-16 - patients (95% CI)	0.4 (0.2, 0.5)	1.6[c] (1.3, 1.8)	3.5[c] (3.0, 4.0)	0.6 (0.3, 0.8)
**Uterine**				
Mean no. patients treated (95% CI), 2007	12 (10, 13)	40[c] (36, 43)	65[c] (59, 72)	16[d] (13, 19)
Mean no. patients treated (95% CI), 2016	15 (13, 18)	62[c] (56, 67)	99[c] (89, 109)	27[c] (22, 32)
Average annual growth rate, 2007-16 - patients (95% CI)	0.4 (0.2, 0.6)	2.4[c] (2.0, 2.8)	3.7[c] (3.0, 4.5)	1.2[c] (0.8, 1.6)
**Pancreatic**				
Mean no. patients treated (95% CI), 2007	4.0 (3.3, 4.8)	13[c] (12, 15)	49[c] (46, 52)	4.8 (3.3, 6.3)
Mean no. patients treated (95% CI), 2016	6.7 (5.5, 7.8)	25[c] (22, 28)	83[c] (78, 88)	9.6[e] (7.1, 12)
Average annual growth rate, 2007-16 - patients (95% CI)	0.3 (0.2, 0.4)	1.3[c] (1.1, 1.5)	3.8[c] (3.4, 4.2)	0.5[e] (0.3, 0.7)

[a]reference
[b]non-Hodgkin Lymphoma, including chronic lymphocytic leukemia/small lymphocytic lymphoma
[c]p<0.001 compared to reference
[d]p=0.02 compared to reference
[e]p=0.03 compared to reference
All others p>0.05

© 2021 Frosch ZAK et al. *JAMA Network Open.*
